# Impact evaluation of invisible intimate partner violence on maternal healthcare utilization in Pakistan

**DOI:** 10.1186/s12884-024-06584-y

**Published:** 2024-05-24

**Authors:** Xinfang Xu, Di Liang, Saeed Anwar, Yanan Zhao, Jiayan Huang

**Affiliations:** 1 Shanghai Institute of Infectious Disease and Biosecurity, School of Public Health, Fudan University, Shanghai, 200032 China; 2 Prime Institute of Public Health, Peshawar Medical College, Peshawar, 25000 Pakistan; 3 Division of Biostatistics, Department of Population Health, New York University School of Medicine, New York, NY 10016 USA

**Keywords:** Intimate partner violence, Maternal healthcare, Emotional violence, Pakistan

## Abstract

**Introduction:**

Existing research has shown that intimate partner violence (IPV) may hinder maternal access to healthcare services, thereby affecting maternal and child health. However, current studies have ignored whether emotional intimate partner violence (EV) could negatively affect maternal healthcare use. This study aims to evaluate the impact of invisible IPV on maternal healthcare utilization in Pakistan.

**Methods:**

We analyzed nationally representative data from the Pakistan Demographic and Health Survey database from 2012–2013 and 2017–2018. Exposure to physical intimate partner violence (PV) and EV was the primary predictor. Based on women’s last birth records, outcomes included three binary variables indicating whether women had inadequate antenatal care (ANC) visits, non-institutional delivery, and lack of postnatal health check-ups. A logistic regression model was established on weighted samples.

**Results:**

Exposure to EV during pregnancy was significantly associated with having inadequate ANC visits (aOR = 2.16, 95% CI: 1.06 to 4.38, *p* = 0.033) and non-institutional delivery (aOR = 2.24, 95% CI: 1.41 to 3.57, *p* = 0.001). Lifetime exposure to EV was associated with increased risks of inadequate ANC visits (aOR = 1.48, 95% CI: 1.00 to 2.19, *p* = 0.049). Lifetime exposure to low-scale physical intimate partner violence (LSPV) (adjusted OR (aOR) = 1.73, 95% CI: 1.29 to 2.31, *p* < 0.001) was associated with increased risks of having no postnatal health check-ups.

**Conclusions:**

Pregnant women who experienced EV and LSPV are at greater risk of missing maternal healthcare, even if the violence occurred before pregnancy. Therefore, in countries with high levels of IPV, early screening for invisible violence needs to be integrated into policy development, and healthcare providers need to be trained to identify EV and LSPV.

**Supplementary Information:**

The online version contains supplementary material available at 10.1186/s12884-024-06584-y.

## Introduction

Intimate partner violence (IPV) refers to behavior within an intimate partner that causes physical, sexual, or psychological harm [1]. While victims of violence include both men and women, the incidence of IPV is much higher in women than in men [2]. For a long time, IPV against women has been a serious global public health problem and human rights abuse [3, 4]. A 2018 study of women aged 15–49 in 161 countries and territories found that globally, it is estimated that 27% of women aged 15–49 with a partner have experienced physical or sexual violence, or both, during their lifetime [5]. Intimate partner violence is not only physical violence in the traditional sense, but also includes many invisible violence types such as emotional violence and low-scale physical violence [6]. Compared with overt physical violence, this two violence are often harder to detect, and thus their impact on the body is often ignored [7].

Women who experience IPV may suffer acute or non-acute health impairments [8] and adverse perinatal health outcomes, such as preterm birth and miscarriage [9]. Some studies suggested that the impact of IPV on maternal and child health is likely to occur through the impact on maternal healthcare utilization [10–12]. Maternal healthcare providers are also in a unique position to spot and help IPV victims. Therefore, maternal healthcare utilization could be a precursor to potential interventions to prevent the impact of IPV on maternal and child health. Although studies have hinted at the impact of IPV on maternal health use, most of them focused on analyzing the impact of aggregated violence on antenatal care [12, 13]. Whether different types of violence have an impact remains open to debate, especially this invisible violence. Moreover, current research has been limited to a few countries in sub-Saharan Africa and South Asia [14–16]. Studies focusing on the impact of neglected intimate partner violence on the overall process of maternal healthcare utilization (including before, during, and after delivery) are still lacking.

As a South Asian country with a high burden of IPV, Pakistan also has a serious problem with maternal morbidity and mortality. According to a systematic review of IPV, the overall prevalence of physical and emotional violence in Pakistan is 10.0–98.5% and 31.3–83.6%, respectively [13]. In 2017, Pakistan had a maternal mortality rate of 140 deaths per 100,000 live births. Meanwhile, Pakistan also suffers from low utilization of maternal healthcare and low quality of maternal healthcare. The USAID study found that about half of pregnant women in Sindh still had fewer than four antenatal care visits in 2013, and about 40 percent of women did not receive postnatal care [17]. Most women also have difficulty finding the right doctor and do not receive any counseling on birth signs, family planning, and danger signs [18].

Current research in Pakistan has focused on the impact of IPV on adverse maternal pregnancy outcomes [19] (e.g., abortion, maternal death, etc.), antenatal care [20], and the location of delivery [21]. Some studies have also analyzed the impact of women’s attitudes toward IPV on maternal healthcare use [22]. However, most of these relevant studies of Pakistan in the past remained localized and didn’t use Pakistan’s nationally representative data.

This study aimed to comprehensively analyze the impact of different types of IPV on maternal health use. Specifically, it focuses on whether invisible intimate partner violence defined as emotional intimate partner violence (EV)and low-scale physical intimate partner violence (LSPV) influences the utilization of maternal healthcare services throughout the course of delivery.

## Methods

### Data source and study sample

To maximize the sample size, we chose two waves (2012–2013, 2017–2018) of the data from the Pakistan Demographic and Health Survey (PDHS). Five DHS studies have been conducted in Pakistan since 1990, and these two waves were the third and fourth survey waves, which were also the latest two surveys that include intimate partner violence data.

The PDHS database, with technical assistance from the National Institute of Population Research (NIPS) and the Pakistan Bureau of Statistics (PBS), focuses on the health of adolescents and women [23]. The survey was conducted using a two-stage stratified random sampling design. The first stage involved selecting sample points (clusters) consisting of enumeration blocks, which is the number of households residing in the enumeration blocks at the time of the census. In the second phase, a sample of households is drawn from a list of households in each selected cluster. Weighted factors have been calculated and added to the data file by PDHS researchers so that the results are representative at the country level (except for Azad Jammu and Kashmir, and Gilgit Baltistan).

**Fig. 1:**
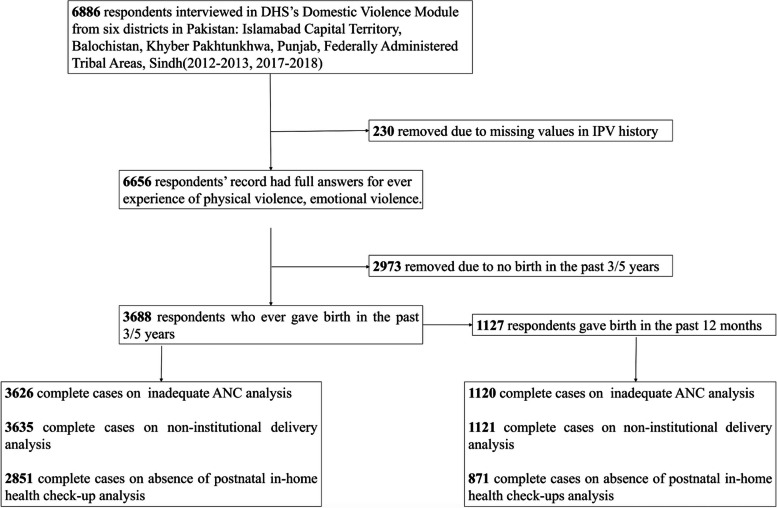
Flowchart diagram of respondents’ records selection process. Note: ANC = antenatal care

In the PDHS 2012–2013 and 2017–2018 database, a total of 8,024 women aged between 15 and 49 participated in the questionnaire survey of the domestic violence module. Due to the lack of weighted data for Azad Jammu and Kashmir, and Gilgit Baltistan in the database, this study removed the data for these areas. Therefore, in this study, 6,886 women from Balochistan, Khyber Pakhtunkhwa (KPK), Punjab, Sindh, Islamabad Capital Territory (I.C.T), and Federally Administered Tribal Areas (F.A.T.A) were selected as the research participants. 6,656 women have completed the questions about physical or emotional violence. Of these women, the study sample was further limited to 3,688 women who had at least one live birth in the past 3/5 years before the start of the survey and 1,127 women who had given birth in the past year. Lifetime exposure and exposure in the past 12 months were divided into three sample groups based on outcome variables; the exact sample size varies from model to model due to the presence of missing values of each outcome variable and control variable. The specific sample size is shown in the flow chart in Fig. 1.

### Measures

#### Primary predictors

In this study, IPV was measured by two sets of predictors which varied in their nature and time frame. According to the domestic violence questionnaire, IPV was first classified as physical intimate partner violence (PV) and EV. Regarding PV, it was measured by a categorical variable indicating no physical violence, LSPV, and severe physical intimate partner violence (SPV). EV was measured by a binary variable indicating the presence of emotional violence. Detailed definitions of each type of IPV are shown in Supplementary Table 1. Our study looked at two-time frames for IPV: lifetime exposure and exposure during pregnancy. To measure IPV during pregnancy, we only included women who had given birth in the previous 12 months.

#### Outcomes

We examined three outcome variables to represent women’s entire process of maternal care utilization before, during, and after their last delivery in the past 3/5 years:

*Having inadequate antenatal care (ANC) visits* is evaluated based on whether a pregnant woman received healthcare less than eight times from a professional health institution [24]. The variable was assigned the value of 0 if the number of healthcare is 8 or more. Otherwise, it was assigned with 1 instead.

*Non-institutional delivery* is defined as the delivery of a pregnant woman, not in a professional delivery facility, where professional delivery facilities include government or private hospitals, clinics, basic health units, rural health centers, and community midwife set up. The results of the survey were converted into a binary output. A value of 0 was assigned if the woman’s last birth before the survey was institutional, and a value of 1 otherwise.

*Having no postnatal check-ups for mothers after delivery* is defined as the absence of any health examination for mothers after discharging from place of delivery. This variable included two questions in the questionnaire, namely whether women who gave birth in institutions had received a health check after discharge and whether women who gave birth outside institutions had received a health check after delivery. The variable was assigned a value of 0 if the woman received a health check after the last delivery before the survey and a value of 1 otherwise.

#### Control variables

The selection of control variables was based on previous research on the influencing factors of IPV and maternal healthcare [25, 26]. The control variables include the age of the woman, the education level of the woman, the working status of the woman (yes or no), the wealth of the family, the year of the survey, women’s decision-making power over healthcare (yes or no), the current residence of the family and the working status of the spouse (yes or no). The ages of the women were divided into four subgroups: 15–23, 24–32, 33–40, and 41–49. The education level of the surveyed women was categorized into four grades according to their highest degree: no education, primary (1–5 grades), secondary (9–10 grades), and higher (year 11 or above). The year of the survey is divided into 2012–2013 and 2017–2018. According to the classification of the DHS, family wealth is assorted into five levels: the poorest, poorer, middle, richer, and richest. The current residence of the family is classified as urban or rural.

### Statistical analysis

In the descriptive analysis, the sociodemographic characteristics of the respondents were presented as unweighted numbers (N) and proportions (%). In addition, the weighted prevalence of IPV in different regions will be presented in the form of a map based on 3303 samples of the PDHS 2017–2018 domestic violence module.

Logistic regression models were applied to examine the relationship between two types of IPV and three outcome variables of maternal healthcare utilization by calculating adjusted odds ratio (aOR) with a 95% confidence interval (95% CI) while controlling for confounding variables.

The logistic regression model for each outcome can be written as:

  

$$\mathrm{logit}\left\{\mathrm{pr}\left({\mathrm Y}_{\mathrm{ijk}}=1\vert{\mathrm X}_{\mathrm{ik},\mathrm{PV}},{\mathrm X}_{\mathrm{ik},\mathrm{EV}},{\mathrm Z}_{\mathrm{ik}}\right)\right\}={\mathrm\alpha}_{0\mathrm{jk}}+{\mathrm\alpha}_{1\mathrm{jk}}{\mathrm X}_{\mathrm{ik},\mathrm{PV}}+{\mathrm\alpha}_{2\mathrm{jk}}{\mathrm X}_{\mathrm{ik},\mathrm{EV}}+{\mathrm Z}_{\mathrm{ik}}^{\mathrm T}\mathrm\beta_{\mathrm k}$$

${\text{X}}_{\text{ik},\text{PV}}$ and ${\text{X}}_{\text{ik},\text{EV}}$ are the PV and EV values of the ${\text{i}}^{\text{th}}$ woman in ${\text{k}}^{\text{th}}$ group, respectively, and ${\text{Z}}_{\text{ik}}$ is a vector containing all control variables of interest. Suppose there are ${\text{n}}_{\text{k}}$ women in the ${\text{k}}^{\text{th}}$ given group, $\text{k}=1, 2$ represent group where women ever gave birth in the past past 5 years and group where women gave birth in the past 12 months, respectively. Let ${\text{Y}}_{\text{ijk}}$ be the ${\text{j}}^{\text{th}}$ outcome values of ${\text{i}}^{\text{th}}$ woman in ${\text{k}}^{\text{th}}$ group, $\text{i}=1, 2, ..., {\text{n}}_{\text{k}},\text{ k}=1, 2$, and $\text{j}=1, 2, 3$ are the signals for three outcome variables, corresponding to inadequate ANC visits, non-institutional delivery, and the absence of postnatal health check-ups for mothers after delivery, respectively.

In this study, we focused on aOR, specifically $\exp({\mathrm\alpha}_{1\mathrm{jk}})$ , and $\exp({\mathrm\alpha}_{2\mathrm{jk}})$ , which quantify the impact of a one-unit change in PV and EV on the ratio of the ${\text{j}}^{\text{th}}$ outcome, allowing for a more accurate assessment of the relationship between event occurrence and EV and PV. Furthermore, a 95% CI and *p*-value for each aOR parameter were calculated using the two sided t-test method. In this study, the Stata survey (svy) commands in STATA were employed to adjust for sampling weight and clustering effects, considering a *p*-value < 0.05 as significant. T statistics were utilized to test the significance of coefficients rather than z statistics due to the application of the svy commands [27]. STATA 17.0 was used to conduct the data analysis.

## Results

### Sample characteristics

**Fig. 2:**
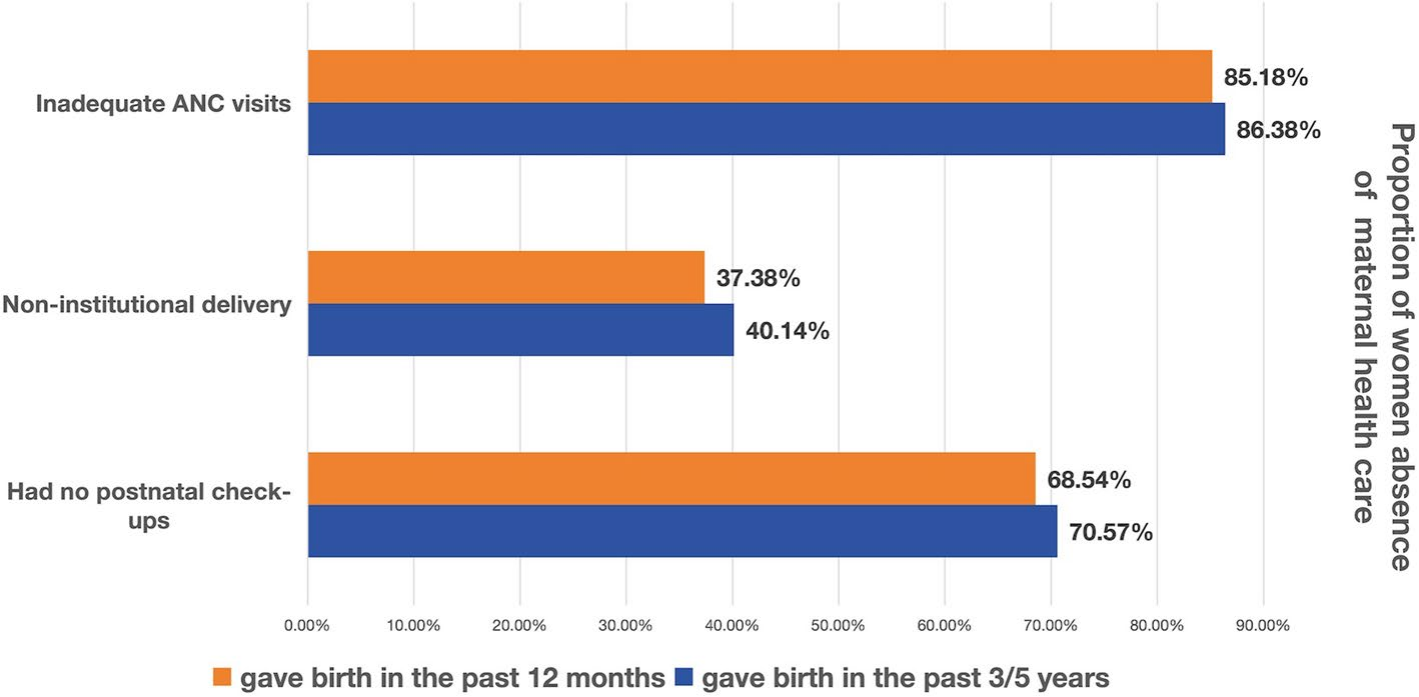
Absence of maternal healthcare utilization among women in the two sampling groups. Note: ANC = antenatal care. Inadequate ANC visits is defined as a pregnant woman received healthcare less than eight times from a professional health institution. Non-institutional delivery is defined as the delivery of a pregnant woman not in a professional delivery facility, where professional delivery facilities include government or private hospitals, clinics, basic health units, rural health center, and community midwife set up

As shown in Fig. 2, the non-utilization rate of maternal healthcare among women in Pakistan was found to be high, particularly for antenatal care and postnatal health check-ups. Of the women who had given birth in the past 3/5 years in our study, 86.38% received less than 8 ANC visits, and 40.14% chose non-institutional delivery. For women who had given birth in the past 12 months, 85.18% had less than 8 ANC visits, and 68.54% did not participate in postnatal health check-ups. Overall, for all three indicators, women who gave birth in the past 3/5 years had a higher inadequate utilization rate of maternal healthcare than women who gave birth in the past 12 months.

In our research, approximately 80% of the participants are in mature years, namely 24 to 40 years old, and half lived in rural religions. More than 50% of whom were shown to have no education and over four out of five respondents were not involved in any work at the time of the survey. Significantly, less than half of women had decision-making power over healthcare (Tables 1 and 2).

**Table 1:** Characteristics of respondents given birth in the past 3/5 years in three maternal healthcare outcome sample groups with unweighted number (N) and proportion (%)

Variables	ANC^a^ visits (*N* = 3626)	Place of delivery^g^ (*N* = 3635)	Postnatal health check-ups (*N* = 2851)
**Experience of IPV**^**b**^** during lifetime**			
** EV**^**c**^			
No	2360 (65.09)	2367 (65.12)	1806 (63.35)
Yes	1266 (34.91)	1268 (34.88)	1045 (36.65)
** PV**^**d**^			
No	2463 (67.93)	2470 (67.95)	1876 (65.80)
LSPV^e^	881 (24.30)	882 (24.26)	734 (25.75)
SPV^f^	282 (7.78)	283 (7.79)	241 (8.45)
** Year of survey**			
2012 to 2013	1813 (50.00)	1814 (49.90)	1033 (36.23)
2017 to 2018	1813 (50.00)	1821 (50.10)	1818 (63.77)
**Individual level factors**			
** Age**			
15–23	587 (16.19)	587 (16.15)	458 (16.06)
24–32	1839 (50.72)	1845 (50.76)	1464 (51.35)
33–40	989 (27.28)	991 (27.26)	757 (26.55)
41–49	211 (5.82)	212 (5.83)	142 (6.03)
** Education level of women**			
No education	1980 (54.61)	1985 (54.61)	1703 (59.73)
Primary	514 (14.18)	515 (14.17)	391 (13.71)
Senior	657 (18.12)	659 (18.13)	451 (15.82)
Higher	475 (13.10)	476 (13.09)	306 (10.73)
** Women current employment status**			
Yes	631 (17.40)	631 (17.36)	493 (17.29)
No	2995 (82.60)	3004 (82.64)	23,584 (82.71)
**Household level factors**			
** Education level of women’s partner**			
No education	1121 (30.92)	1124 (30.92)	970 (34.02)
Primary	528 (14.56)	529 (14.55)	434 (15.22)
Senior	1177 (32.46)	1177 (32.38)	903 (31.67)
Higher	800 (22.06)	805 (22.15)	544 (19.08)
** Wealth index**			
Poorest	812 (22.39)	814 (22.39)	734 (25.75)
Poorer	762 (21.01)	764 (21.02)	665 (23.33)
Middle	545 (17.79)	645 (17.74)	533 (18.70)
Richer	725 (19.99)	728 (20.03)	515 (18.06)
Richest	682 (18.81)	684 (18.82)	404 (14.17)
** Place of residence**			
Urban	1614 (44.51)	1619 (44.54)	1200 (42.09)
Rural	2012 (55.49)	2016 (55.46)	1651 (57.91)
** Decision Power in healthcare**			
Yes	1665 (45.92)	1668 (45.89)	1251 (43.88)
No	1961 (54.08)	1967 (54.11)	1600 (56.12)

Variables included predictive variables (levels of intimate partner violence), individual level factors, household level factors and database year

^a^*ANC* Antenatal care

^b^*IPV* Intimate partner violence

^c^*EV* Emotional intimate partner violence

^d^*PV* Physical intimate partner violence

^e^*LSPV* Low-scale physical intimate partner violence

^f^*SPV* Severe physical intimate partner violence

^g^Place of delivery is divided into institutional and non-institutional delivery

**Table 2:** Characteristics of respondents given birth in the past 12 months in three maternal healthcare outcome sample groups with unweighted number (N) and proportion (%)

Variables	ANC^a^ visits (*N* = 1120)	Place of delivery^g^ (*N* = 1121)	Postnatal health check-ups (*N* = 871)
**Experience of IPV**^**b**^** during pregnancy**			
** EV**^**c**^			
No	807 (72.05)	808 (72.08)	611 (70.15)
Yes	313 (17.95)	313 (27.92)	260 (29.85)
** PV**^**d**^			
No	884 (78.93)	885 (78.95)	681 (78.19)
LSPV^e^	186 (16.61)	186 (16.59)	148 (16.99)
SPV^f^	50 (4.46)	50 (4.46)	42 (4.82)
** Year of survey**			
2012 to 2013	494 (44.11)	567 (50.58)	318 (36.51)
2017 to 2018	626 (55.89)	554 (49.42)	553 (63.48)
**Individual level factors**			
** Age**			
15–23	286 (25.54)	286 (25.51)	222 (25.49)
24–32	602 (53.75)	602 (53.70)	467 (53.62)
33–40	211 (18.84)	211 (18.82)	161 (18.48)
41–49	21 (1.88)	22 (1.96)	21 (2.41)
** Education level of women**			
No education	586 (52.32)	587 (52.36)	501 (57.52)
Primary	162 (14.46)	162 (14.45)	117 (13.43)
Senior	214 (19.11)	214 (19.09)	144 (16.53)
Higher	158 (14.11)	158 (14.09)	109 (12.52)
** Women current employment status**			
Yes	165 (14.73)	165 (14.72)	136 (15.61)
No	955 (85.27)	956 (85.28)	735 (84.39)
**Household level factors**			
** Education level of women’s partner**			
No education	325 (29.02)	326 (29.08)	273 (31.34)
Primary	169 (15.09)	169 (15.08)	136 (15.61)
Senior	368 (32.86)	368 (32.83)	284 (32.61)
Higher	258 (23.04)	258 (23.02)	178 (20.44)
** Wealth index**			
Poorest	239 (21.34)	240 (21.41)	214 (24.57)
Poorer	247 (22.05)	247 (22.03)	211 (24.23)
Middle	196 (17.50)	196 (17.48)	160 (18.37)
Richer	231 (20.63)	231 (20.61)	158 (18.14)
Richest	207 (18.48)	207 (18.47)	128 (14.69)
** Place of residence**			
Urban	494 (44.11)	494 (44.07)	365 (41.91)
Rural	626 (55.89)	627 (55.93)	506 (58.09)
** Decision Power in healthcare**			
Yes	482 (43.04)	482 (43.00)	368 (42.25)
No	638 (56.96)	639 (57.00)	503 (57.75)

Variables included predictive variables (levels of intimate partner violence), individual level factors, household level factors and database year

^a^*ANC* Antenatal care

^b^*IPV* Intimate partner violence

^c^*EV* Emotional intimate partner violence

^d^*PV* Physical intimate partner violence

^e^*LSPV* Low-scale physical intimate partner violence

^f^*SPV* Severe physical intimate partner violence

^g^Place of delivery is divided into institutional and non-institutional delivery

### Prevalence of IPV in Pakistan

**Fig. 3:**
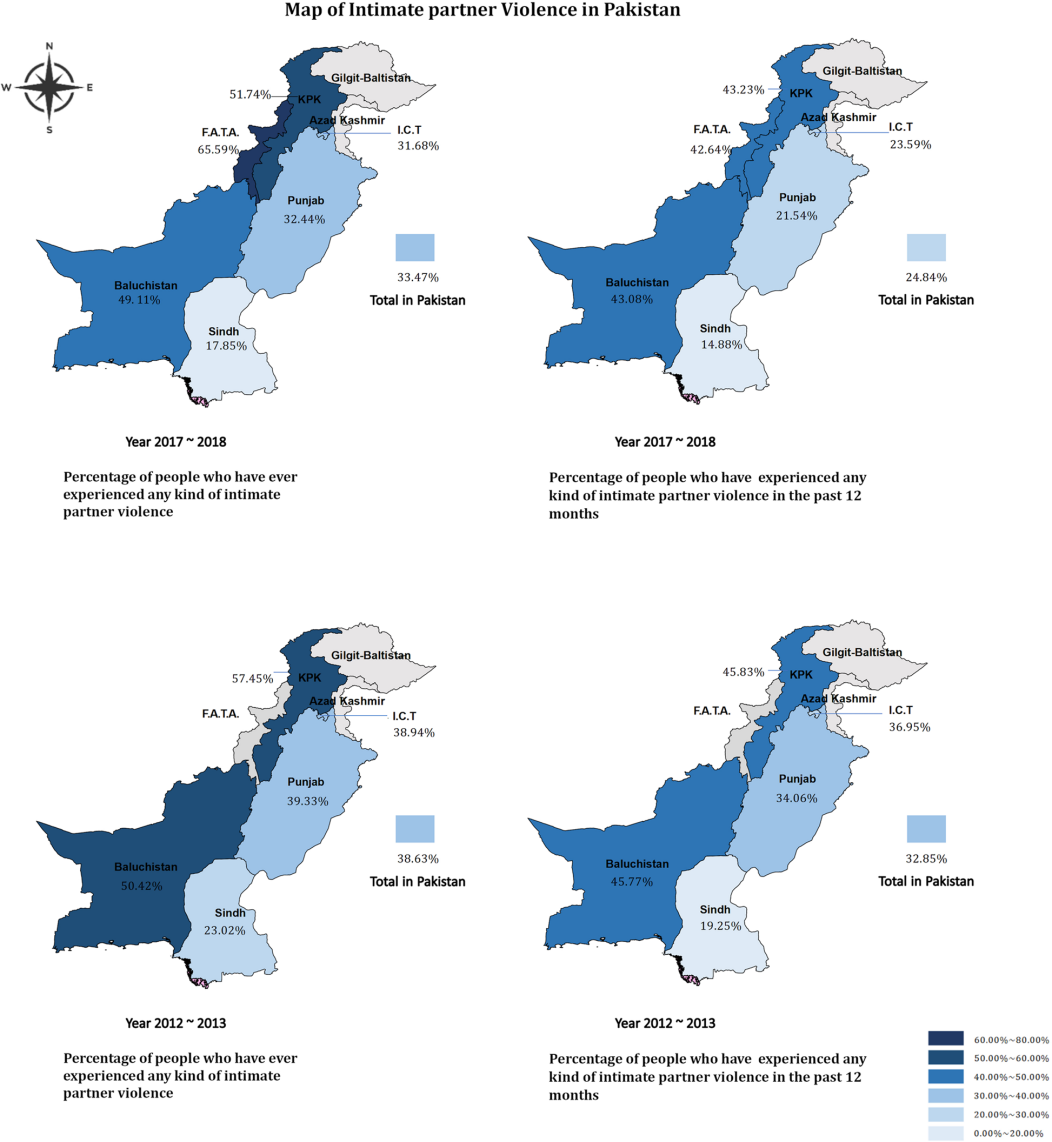
Prevalence of any types of intimate partner violence in Pakistan in year 2012–2013 and 2017–2018. Note: F.A.T. A = Federally Administered Tribal Areas, KPK = Khyber Pakhtunkhwa, I.C.T = Islamabad Capital Territory. F.A.T.A data were not included in the PDHS2012-2013 data, so prevalence rates were listed for only five administrative divisions in year 2012–2013

Figure 3 presents the prevalence of any type of IPV (both PV and EV) in the different administrative divisions of Pakistan in 2012–2013 and 2017–2018. The overall lifetime prevalence of IPV in Pakistan decreased from 38.63% in 2012–2013 to 33.47% in 2017–2018, with a downward trend across all regions (Fig. 3). Specific figures on the prevalence of PV and EV in different regions during these two periods are shown in Supplement Fig. 1 and Fig. 2.

Overall, the distribution of each type of violence follows a similar pattern, where the northwest region of Pakistan has a higher prevalence of IPV against women. This indicates a consistent pattern between 2012–2013 and 2017–2018. In the following description, we use 2017–2018 as an example to precisely describe the prevalence of IPV in different regions of Pakistan. Specifically, F.A.T.A has the highest victim proportions among all the administrative divisions, with 65.59% of women having experienced IPV during their lifetime (Fig. 3); among them, 42.25% and 64.51% have ever experienced LSPV, and EV respectively in the year 2017–2018 (supplement Fig. 1).

The prevalence of IPV in the past 12 months was slightly lower than the lifetime prevalence, but it also shows a pattern of higher prevalence in the northwest than in the southeast. Overall, the KPK had the highest prevalence of intimate partner violence in the past 12 months (45.83% in year 2012–2013 and 43.23% in year 2017–2018). The prevalence of IPV in other areas is shown in Fig. 3.

### Impact of IPV on maternal healthcare

Estimates of the logistic analysis of IPV and maternal healthcare utilization among women after adjusting for confounders are presented in Tables 3 and 4. Results show that pregnant women’s experience of EV and LSPV is a strong predictor of their absence in maternal healthcare before and after delivery.

**Table 3:** Associations between maternal exposure to different forms of IPV^a^ and use of maternal healthcare services during lifetime

Variables	Had less than 8 ANC^b^ visits	Had non-institutional delivery	Had no postnatal health check-ups
	**aOR (95% CI)** ***P* value**	**aOR (95% CI)** ***P* value**	**aOR (95% CI)** ***P* value**
**Experience of EV**^**c**^** during lifetime**			
No	[REF]	[REF]	[REF]
Yes	1.48 (1.00 to2.19) *p* = 0.049	1.16 (0.89 to 1.50) *p* = 0.281	1.11 (0.82 to 1.51) *P* = 0.493
**Experience of PV**^**d**^** during lifetime**			
No	[REF]	[REF]	[REF]
LSPV^e^	1.15 (0.74 to 1.78) *p* = 0.533	1.06 (0.80 to 1.39) *p* = 0.691	1.73 (1.29 to 2.31) *p* < 0.001
SPV^f^	0.87 (0.31 to 2.42) *p* = 0.787	1.01 (0.68 to 1.50) *p* = 0.949	1.25 (0.78 to 1.98) *P* = 0.352
Weighted N	3768	3776	2909

The analyzed data are from two data sets PDHS 2012–2013 and PDHS 2017–2018. PV is divided into low-scale physical violence and severe physical violence as a rank variable

Models adjusted for mother’s age, mother’s education, mother’s work status, partner’s education, family wealth status, women's decision-making power over healthcare, place of residence, year of survey, and types of IPV

^a^*IPV* Intimate partner violence

^b^*ANC* Antenatal care

^c^*EV* Emotional intimate partner violence

^d^*PV* Physical intimate partner violence

^e^*LSPV* Low-scale physical intimate partner violence

^f^*SPV* Severe physical intimate partner violence

**Table 4:** Associations between maternal exposure to different forms of IPV^a^ during pregnancy and use of maternal healthcare services

Variables	Had Less than 8 ANC^b^ visits	Had non-institutional delivery	Had no postnatal health check-ups
	**aOR (95% CI)** ***P* value**	**aOR (95% CI)** ***P* value**	**aOR (95% CI)** ***P* value**
**Experience of EV**^**c**^** during pregnancy**			
No	[REF]	[REF]	[REF]
Yes	2.16 (1.06 to 4.38) *p* = 0.033	2.24 (1.41 to 3.57) *p* = 0.001	1.14 (0.70 to 1.87) *p* = 0.676
**Experience of PV**^**d**^** during pregnancy**			
No	[REF]	[REF]	[REF]
LSPV^e^	1.65 (0.51 to 5.27) *p* = 0.400	0.59 (0.33 to 1.08) *p* = 0.085	1.52 (0.79 to 2.94) *p* = 0.303
SPV^f^	0.56 (0.15 to 2.07) *p* = 0.383	0.53 (0.22 to 1.31) *p* = 0.170	0.95 (0.34 to 2.67) *p* = 0.522
Weighted N	1235	1235	1032

The analyzed data are from two data sets PDHS 2012–2013 and PDHS 2017–2018. PV is divided into low-scale physical violence and severe physical violence as a rank variable

Models adjusted for mother’s age, mother’s education, mother’s work status, partner’s education, family wealth status, women's decision-making power over healthcare, place of residence, year of survey, and types of IPV

^a^*IPV* Intimate partner violence

^b^*ANC* Antenatal care

^c^*EV* Emotional intimate partner violence

^d^*PV* Physical intimate partner violence

^e^*LSPV* Low-scale physical intimate partner violence

^f^*SPV* Severe physical intimate partner violence

According to the findings, pregnant women who experienced EV were at greater risk of missing antenatal care and institutional delivery, even if the violence occurred before pregnancy. Exposure to EV during pregnancy significantly impacted adequate ANC visits and institutional delivery. The odds of inadequate ANC visits and non-institutional delivery were 2.16 times (95% CI: 1.06 to 4.38, *p* = 0.033) and 2.24 times (95% CI: 1.41 to 3.57, *p* = 0.001), respectively higher for those women who experienced EV than women who had not experienced it (Table 4). At the same time, lifetime EV continued to affect ANC. The odds of inadequate ANC visits were 1.48 times higher (95% CI: 1.00 to 2.19, *p* = 0.049) for women who experienced EV during their lifetime than women who had not experienced it (Table 3).

Another variable that affects maternal health use is low-scale PV. However, the impact of lifetime exposure to low-scale PV on maternal healthcare slightly differs from that experienced in the past 12 months. According to the results, lifetime exposure to low-scale PV significantly reduces the likelihood of a mother having a postnatal health check-up. The odds of no postnatal health check-ups were 1.73 times higher (95% CI: 1.29 to 2.31, *p* < 0.001) for women who experienced low-scale PV during their lifetime than women who had not experienced it (Table 3). The odds ratio for women who experienced PV (both low-scale and severe) during pregnancy was statistically not significant, indicating no difference between participation or non-participation in maternal healthcare (Table 4).

## Discussions

Using data from PDHS 2012–2013 and 2017–2018, we found that Pakistan has a high rate of IPV, with EV and LSPV being the dominant types. Our study found that low-scale PV and EV can hurt maternal health use. Two of the most significant results were that lifetime exposure to LSPV reduced women’s likelihood of using postnatal health check-ups, while exposure to EV within the last 12 months had a strong negative impact on having adequate ANC visits and institutional delivery. According to our findings, invisible IPV could harm all aspects of maternal healthcare, while SPV does not affect access to maternal healthcare. The results are consistent with studies that have found that LSPV and EV may affect antenatal care utilization and institutional delivery negatively [28, 29]. However, SPV, which has been shown to significantly decrease the likelihood of adequate ANC visits and institutional delivery in some previous studies [21], did not show significant results in our study. Our study did not find an effect of SPV on the inadequate utilization of maternal healthcare, which may be due to the relatively small sample sizes of the subgroup in our study with exposure to SPV.

Our research found that EV during pregnancy, while often overlooked, has a significant impact on maternal healthcare utilization and therefore on maternal and infant health. EV is one of the most common types of IPV but is often overlooked because of its insidious nature [30]. In previous studies, lifelong exposure to EV has been found to correlate with whether an institutional birth was chosen, which is consistent with the results of this study. However, the slight difference is that previous studies have considered less than 4 times as inadequate ANC visits; In this study, according to the latest standards of WHO, less than 8 times were considered as insufficient ANC visits. Our research further focused on EV during pregnancy and found that exposure to EV during pregnancy not only affected the location of delivery but also the amount of antenatal care a woman received. Compared with PV, we found that EV has a significant impact on maternal healthcare utilization, which affects both antenatal and childbirth. Although EV is often accompanied by PV, the impact of EV on pregnant women’s access to antenatal care and institutional delivery remains significant even after we exclude the influence of PV. In contrast, PV only affects the postnatal period. A possible reason is that EV often has a profound impact on women’s self-esteem and mental health, especially pregnant women. This emotional damage can also represent an unsupportive, neglected attitude of a spouse towards pregnant women, making it difficult for women to take the initiative to seek maternal healthcare in Pakistan, where women are generally poorly educated.

The study also found that exposure to LSPV was associated with a decrease in postnatal healthcare. This negative effect may be due to the avoidance of health checks by women who have experienced LSPV [31]. Controlling behavior from partners may also limit healthcare providers’ access to home [32]. Some studies outside Pakistan have also found that PV can have a negative effect on postnatal healthcare [29, 31]. However, these studies did not break down the severity of PV, so it is difficult to explain whether different levels of PV have different effects. Compared with SPV, LSPV often does not cause visible harm to the body itself but can undermine maternal initiative in accessing health services and ultimately affect postnatal health [33]. This kind of violence usually occurs at home or in other private places, and because the impact is small, women often choose to lie to cover it up. This is also a possible reason why LSPV can interfere with postnatal check-ups since husbands tend to be reluctant to be found out about their violent behavior.

The above results and discussions suggest the impact of EV and LSPV on maternal healthcare use and provide new ideas for improving maternal healthcare use. Religious beliefs in Pakistan exert significant influence on women’s health-related decisions [34]. The gender inequality stemming from a patriarchal society, coupled with low education levels, contributes to high rates of IPV [35] and limits women’s access to maternal healthcare. Similar patterns of IPV exist in other South Asian countries. Therefore, our findings can not only provide a solid policy basis for Pakistan, but also provide valuable insights for other countries in South Asia, especially countries with cultural backgrounds similar to Pakistan.

Governments and other stakeholders need to work to change social barriers to gender inequality and reduce the incidence of IPV. Given that EV during pregnancy often goes undetected, health authorities should attach importance to the psychological abuse of women in their interventions, incorporating women’s self-reports and clinical findings. At the same time, the prevention of IPV should also be included in maternal and child health planning to reduce the impact of IPV on maternal health. There should be more screening for EV during pregnancy, and more attention should be paid to invisible intimate partner violence. For mothers, the number of home visits should be increased to reduce the probability of giving up postnatal health check-ups due to LSPV.

### Study strength and limitations

Our study analyzed the impact of IPV on three components of maternal health use in Pakistan, considering both lifetime exposure and exposure during pregnancy. Nationally representative weighted data are used in this study, so the results represent women of childbearing age in Pakistan as a whole. They can provide a reference for other countries in South Asia.

However, our analysis for IPV and maternal healthcare service utilization is based on retrospective cross-sectional survey data, so recall bias is possible. Because the DHS database does not have data on EV during pregnancy, we measured the incidence of intimate partner violence during pregnancy by limiting the approximate time period. This measurement has no exact time limit and is an approximate estimate. At the same time, many women may be reluctant to disclose IPV during the survey process, leading to a lower estimated prevalence of IPV than the actual prevalence.

## Conclusion

Pregnant women who experienced EV and LSPV are at greater risk of missing maternal healthcare, even if the violence occurred before pregnancy. Therefore, in countries with high levels of intimate partner violence, early screening for invisible violence needs to be integrated into policy development, and healthcare providers need to be built up to screen for EV and LSPV.

## Supplementary Information

Supplementary Material 1.

## Data Availability

The survey used data from the Pakistan Demographic and Health Survey (PDHS). Data is available at https://www.dhsprogram.com/data/available-datasets.cfm.

## Abbreviations

IPV: Intimate partner violence
EV: Emotional intimate partner violence
PV: Physical intimate partner violence
LSPV: Low-scale physical intimate partner violence
SPV: Severe physical intimate partner violence
PDHS: Pakistan Demographic and Health Survey
NIPS: National Institute of Population Research
PBS: Pakistan Bureau of Statistics
F. A. T. A: Federally Administered Tribal Areas
ANC: Antenatal care
