# Supplementary material for: Impact evaluation of invisible intimate partner violence on maternal healthcare utilization in Pakistan
Source: BMC Pregnancy Childbirth. 2024 May 24;24:386. doi: 10.1186/s12884-024-06584-y (PMC11636843; doi:10.1186/s12884-024-06584-y)
Supplement: Supplementary file 1 — Supplementary Material 1. [file 12884_2024_6584_MOESM1_ESM.docx]

***Supplementary Material***

**Impact of invisible intimate partner violence on maternal healthcare utilization：**

**A cross-sectional study based on Pakistan Demographic Health Surveys**

**Figure and table legends**

**Supplementary Table 1. Classification of intimate relationship violence based on specific behaviors of violence by spouse**

**Supplementary Table 2. Prevalence of intimate relationship violence and maternal health use among women in two-year groups (2012-2013 and 2017-2018)**

**Note:**

**ANC = Antenatal Care.**

**Supplementary Table 3. Associations between maternal exposure to different forms of IPV, related control variables and use of maternal healthcare services in the past 12 months**

**Supplementary Table 4. Associations between maternal exposure to different forms of IPV, related control variables and use of maternal healthcare services in the past 3/5 years**

***Supplementary Figure 1. Prevalence of emotional violence and physical violence (both severe and low scale) in Pakistan in year 2017-2018**

***Supplementary Figure 2. Prevalence of emotional violence and physical violence (both severe and low scale) in Pakistan in year 2012-2013**

**Note:**

**FATA data were not included in the PDHS2012-2013 data, so prevalence rates were listed for only five administrative divisions**

**Supplementary Table 1. Classification of intimate relationship violence based on specific behaviors of violence by spouse**

| **Types of intimate partner violence** | | **Partner’s behaviors** |
| --- | --- | --- |
| **Emotional violence** | | **Spouse ever humiliated her.** |
|  |  | **Spouse ever threatened her with harm.** |
|  |  | **Spouse ever insulted her or made her feel bad.** |
| **Physical violence** | **Low scale physical violence (mild)** | **Spouse ever pushed, shook or threw something.** |
|  |  | **Spouse ever slapped.** |
|  |  | **Spouse ever punched with fist or something harmful.** |
|  |  | **Spouse ever twisted her arm or pulled her hair.** |
|  | **Severe physical violence** | **Spouse ever kicked or dragged.** |
|  |  | **Spouse ever tried to strangle or burn.** |
|  |  | **Spouse ever threatened with knife/gun or other weapon.** |

**Supplementary Table 2. Prevalence of intimate relationship violence and maternal health use among women in two-year groups (2012-2013 and 2017-2018)**

|  | **PDHS2012-2013** | **PDHS2017-2018** |
| --- | --- | --- |
| **Prevalence of intimate partner violence during lifetime (%)** | | |
| **Emotional violence** | 32.32 | 25.84 |
| **Physical violence** | | |
| severe | 6.66 | 5.47 |
| Mild | 20.36 | 17.45 |
| **Prevalence of intimate partner violence in the past 12 months (%)** | | |
| **Emotional violence** | 28.36 | 20.62 |
| **Physical violence** | | |
| severe | 3.74 | 3.41 |
| Mild | 14.33 | 10.24 |
| **Maternal healthcare utilisation for women who gave birth in the past 3/5 years**  **N (%)** | | |
| Inadequate ^a^ANC visits | 1551 (85.55) | 1581 (87.20) |
| Non-institutional delivery | 846 (46.64) | 613 (33.66) |
| Absence of postnatal check-ups | 756 (73.18) | 1256 (69.09) |
| **Maternal healthcare utilisation for women who gave birth in the past 12 months**  **N (%)** | | |
| Inadequate ANC visits | 482 (97.57) | 472 (75.40) |
| Non-institutional delivery | 255 (44.97) | 164 (29.60) |
| Absence of postnatal check-ups | 224 (70.44) | 373 (67.45) |

Note:

^a^ANC = Antenatal Care.

**Supplementary Table 3. Associations between maternal exposure to different forms of IPV, related control variables and use of maternal healthcare services in the past 12 months**

| **Variables** | **Had Less than 8 ^a^ANC visits** | **Had No facility Delivery** | **Had no postnatal check-ups** |
| --- | --- | --- | --- |
|  | **aOR (95% CI)** | **aOR (95% CI)** | **aOR (95% CI)** |
| **Experienced Emotional violence in the past 12 months** | | |  |
| **No** | [REF] | [REF] | [REF] |
| **Yes** | 2.16  (1.06 to 4.38) * | 2.24  (1.41 to 3.57) ** | 1.14  (0.70 to 1.87) |
| **Experienced Physical violence in the past 12 months** | | |  |
| **No** | [REF] | [REF] | [REF] |
| **Mild** | 1.65  (0.51 to 5.27) | 0.59  (0.33 to 1.08) | 1.52  (0.79 to 2.94) |
| **Severe** | 0.56  (0.15 to 2.07) | 0.53  (0.22 to 1.31) | 0.95  (0.34 to 2.67) |
| **Age group** |  |  |  |
| 15-23 | [REF] | [REF] | [REF] |
| 24-32 | 0.64  (0.34 to 1.20) | 1.12  (0.72 to 1.71) | 1.02  (0.58 to 1.75) |
| 33-40 | 0.92  (0.38 to 2.25) | 0.98  (0.54 to 1.79) | 1.08  (0.55 to 2.13) |
| 41-49 | 7.23  (0.77 to 67.58) | 1.29  (0.34 to 4.98) | 1.69  (0.23 to 12.68) |
| **Education level of women** | | |  |
| Higher | [REF] | [REF] | [REF] |
| Senior | 2.05  (1.01 to 4.18) * | 1.45  (0.51 to 4.16) | 1.14  (0.56 to 2.35) |
| Primary | 2.97  (1.20 to 7.35) * | 1.64  (0.54 to 4.97) | 2.18  (0.91 to 5.19) |
| No education | 5.97  (2.41 to 14.76) *** | 2.87  (0.99 to 8.26) | 2.04  (0.90 to 4.59) |
| **Women current employment status** | | |  |
| Yes | [REF] | [REF] | [REF] |
| No | 1.62  (0.73 to 3.59) | 0.75  (0.44 to 1.27) | 1.29  (0.73 to 2.26) |
| **Education level of women’s partner** | | |  |
| Higher | [REF] | [REF] | [REF] |
| Senior | 0.81  (0.39 to 1.68) | 1.43  (0.74 to 2.76) | 0.94  (0.47 to 1.88) |
| Primary | 1.12  (0.41 to 3.04) | 1.76  (0.81 to 3.81) | 1.01  (0.49 to 2.09) |
| No education | 1.23  (0.48 to 3.15) | 2.29  (1.16 to 4.53) * | 1.11  (0.50 to 2.48) |
| **Wealth index** | | |  |
| Richest | [REF] | [REF] | [REF] |
| Richer | 1.63  (0.72 to 3.72) | 1.66  (0.70 to 3.95) | 1.08  (0.50 to 2.34) |
| Middle | 2.82  (1.04 to 7.67) * | 2.61  (1.06 to 6.44) * | 0.94  (0.41 to 2.12) |
| Poorer | 3.36  (1.13 to 10.03) * | 3.65  (1.48 to 9.01) ** | 1.12  (0.45 to 2.80) |
| Poorest | 6.06  (1.55 to 23.76) ** | 3.51  (1.31 to 9.38) * | 0.80  (0.28 to 2.29) |
| **Place of residence** | | |  |
| Urban | [REF] | [REF] | [REF] |
| Rural | 1.13  (0.60 to 2.14) | 0.91  (0.55 to 1.53) | 1.66  (1.00 to 2.74) * |
| **Decision Power in healthcare** | | |  |
| Yes | [REF] | [REF] | [REF] |
| No | 1.10  (0.68 to 1.76) | 0.80  (0.53 to 1.20) | 1.80  (1.16 to 2.80) ** |
| **Year of survey** | | |  |
| 2012-2013 | [REF] | [REF] | [REF] |
| 2017-2018 | 0.83  (0.49 to 1.39) | 0.35  (0.23 to 0.52) *** | 0.90  (0.59 to 1.40) |
| Weighted N | 1235 | 1235 | 1032 |

Note:

*p<0.05, **p<0.01，***p<0.001

^a^ANC = Antenatal Care.

The analyzed data are from two data sets PDHS2012-2013 and PDHS2017-2018.Physical violence are divided into low scale physical violence and severe physical violence as a rank variable.

**Supplementary Table 4. Associations between maternal exposure to different forms of IPV, related control variables and use of maternal healthcare services in the past 3/5 years**

| **Variables** | **Had Less than 8 ^a^ANC visits** | **Had No facility Delivery** | **Had no Postnatal check-ups** |
| --- | --- | --- | --- |
|  | **aOR (95% CI)** | **aOR (95% CI)** | **aOR (95% CI)** |
| **Ever experienced Emotional violence during lifetime** | | |  |
| **No** | [REF] | [REF] | [REF] |
| **Yes** | 1.48  (1.00 to 2.19) * | 1.16  (0.89 to 1.50) | 1.11  (0.82 to 1.51) |
| **Ever experienced Physical violence during lifetime** | | |  |
| **No** | [REF] | [REF] | [REF] |
| **Mild** | 1.15  (0.74 to 1.78) | 1.06  (0.80 to 1.39) | 1.73  (1.29 to 2.31) ** |
| **Severe** | 0.87  (0.31 to 2.42) | 1.01  (0.68 to 1.50) | 1.25  (0.78 to 1.98) |
| **Age group** |  |  |  |
| 15-23 | [REF] | [REF] | [REF] |
| 24-32 | 0.75  (0.48 to 1.19) | 1.14  (0.82 to 1.58) | 1.02  (0.70 to 1.47) |
| 33-40 | 0.96  (0.58 to 1.57) | 1.06  (0.75 to 1.50) | 1.09  (0.72 to 1.63) |
| 41-49 | 0.71  (0.35 to 1,46) | 1.62  (1.04 to 2.52) * | 1.06  (0.57 to 1.97) |
| **Education level of women** | | |  |
| Higher | [REF] | [REF] | [REF] |
| Senior | 1.81  (1.20 to 2.783) ** | 2.10  (1.17 to 3.78) * | 1.08  (0.69 to 1.67) |
| Primary | 4.17  (2.38 to 7.29) *** | 3.35  (1.78 to 6.31) *** | 1.16  (0.68 to 1.98) |
| No education | 5.35  (3.21 to 8.92) *** | 3.73  (2.14 to 6.53) *** | 1.80  (0.68 to 2.25) |
| **Women current employment status** | | |  |
| Yes | [REF] | [REF] | [REF] |
| No | 1.10  (0.73 to 1.68) | 0.79  (0.59 to 1.04) | 1.17  (0.84 to 1.62) |
| **Education level of women’s partner** | | |  |
| Higher | [REF] | [REF] | [REF] |
| Senior | 0.79  (0.52 to 1.19) | 1.26  (0.88 to 1.79) | 1.21  (0.81 to 1.81) |
| Primary | 0.88  (0.50 to 1.56) | 1.81  (1.22 to 2.68) ** | 1.23  (0.75 to 2.02) |
| No education | 1.21  (0.70 to 2.09) | 1.90  (1.31 to 2.75) *** | 0.88  (0.56 to 1.37) |
| **Wealth index** | | |  |
| Richest | [REF] | [REF] | [REF] |
| Richer | 1.70  (1.09 to 2.65) * | 2.34  (1.42 to 3.85) *** | 1.27  (0.79 to 2.02) |
| Middle | 2.11  (1.22 to 3.66) ** | 2.98  (1.79 to 4.98) *** | 1.41  (0.85 to 2.37) |
| Poorer | 3.68  (1.83 to 7.37) *** | 5.02  (2.99 to 8.42) *** | 1.71  (0.99 to 2.95) |
| Poorest | 5.21  (2.23 to 12.14) *** | 5.40  (2.97 to 9.83) *** | 1.24  (0.68 to 2.25) |
| **Place of residence** | | |  |
| Urban | [REF] | [REF] | [REF] |
| Rural | 1.58  (1.05 to 2.40) * | 1.09  (0.81 to 1.45) | 1.59  (1.17 to 2.16) ** |
| **Decision Power in healthcare** | | |  |
| Yes | [REF] | [REF] | [REF] |
| No | 1.12  (0.84 to 1.50) | 1.06  (0.85 to 1.33) | 1.22  (0.95 to 1.56) |
| **Year of survey** | | |  |
| 2012-2013 | [REF] | [REF] | [REF] |
| 2017-2018 | 1.05  (0.76 to 1.45) | 0.42  (0.33 to 0.54) *** | 0.98  (0.74 to 1.30) |
| Weighted N | 3768 | 3776 | 2909 |

Note:

*p<0.05, **p<0.01，***p<0.001

^a^ANC = Antenatal Care.

The analyzed data are from two data sets PDHS2012-2013 and PDHS2017-2018.Physical violence are divided into low scale physical violence and severe physical violence as a rank variable.


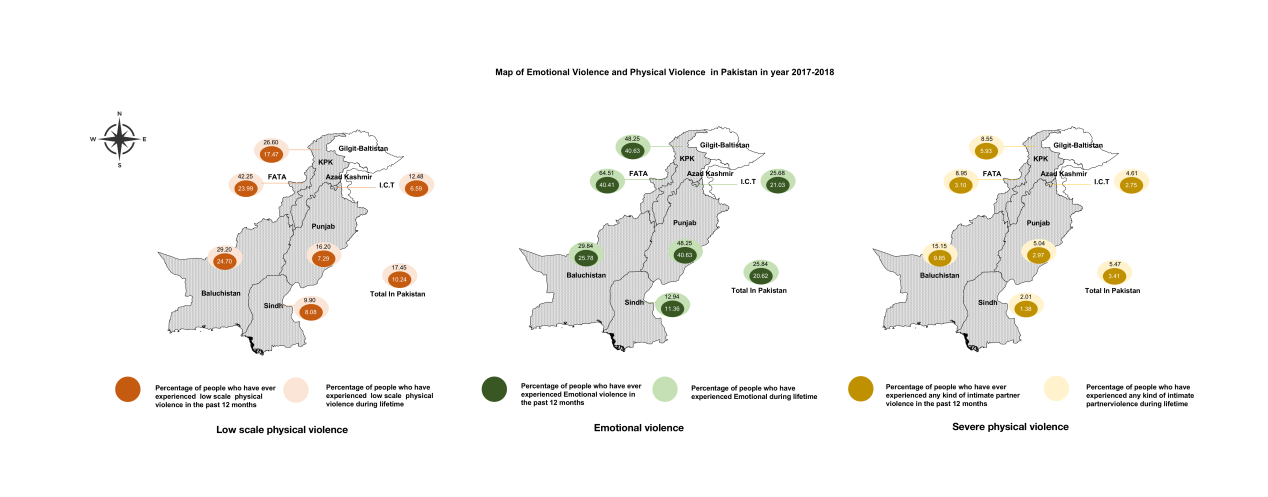


**Supplementary Figure 1. Prevalence of emotional violence and physical violence (both severe and low scale) in Pakistan in year 2017-2018**


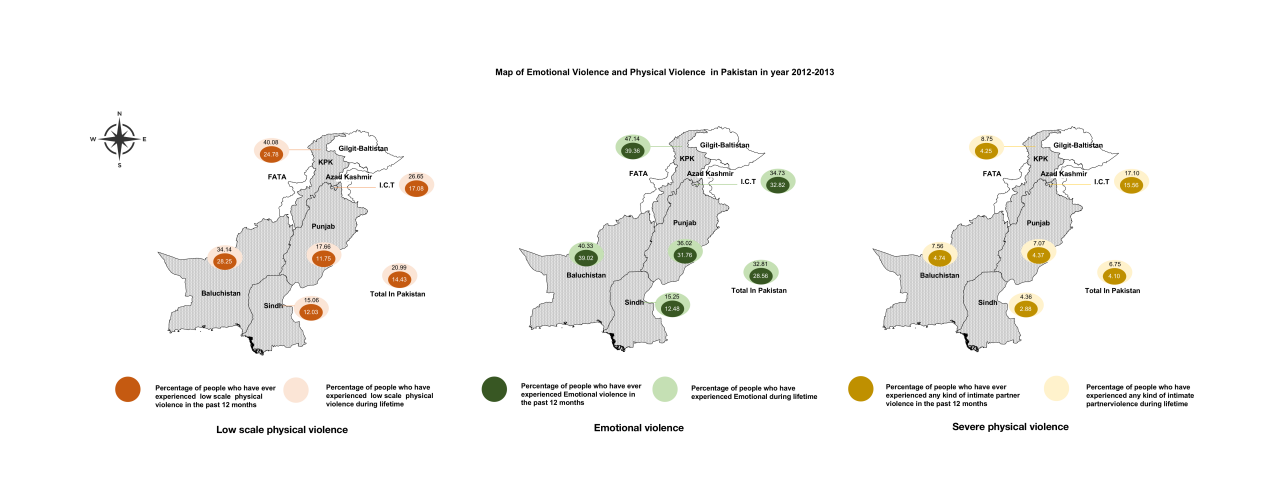


**Supplementary Figure 2. Prevalence of emotional violence and physical violence (both severe and low scale) in Pakistan in year 2012-2013**

**Note:**

**FATA data were not included in the PDHS2012-2013 data, so prevalence rates were listed for only five administrative divisions**
